# Global soil, landuse, evapotranspiration, historical and future weather databases for SWAT Applications

**DOI:** 10.1038/s41597-019-0282-4

**Published:** 2019-11-06

**Authors:** K. C. Abbaspour, S. Ashraf Vaghefi, H. Yang, R. Srinivasan

**Affiliations:** 1 Eawag, Swiss Federal Institute of Aquatic Science and Technology, 8600 Dübendorf, Switzerland; 2 Department of Ecosystem Science and Management, Texas A & M University, College Station, TX 77843 USA

**Keywords:** Climate sciences, Hydrology

## Abstract

Large-scale distributed watershed models are data-intensive, and preparing them consumes most of the research resources. We prepared high-resolution global databases of soil, landuse, actual evapotranspiration (AET), and historical and future weather databases that could serve as standard inputs in Soil and Water Assessment Tool (SWAT) models. The data include two global soil maps and their associated databases calculated with a large number of pedotransfer functions, two landuse maps and their correspondence with SWAT’s database, historical and future daily temperature and precipitation data from five IPCC models with four scenarios; and finally, global monthly AET data. Weather data are 0.5° global grids text-formatted for direct use in SWAT models. The AET data is formatted for use in SWAT-CUP (SWAT Calibration Uncertainty Procedures) for calibration of SWAT models. The use of these global databases for SWAT models can speed up the model building by 75–80% and are extremely valuable in areas with limited or no physical data. Furthermore, they can facilitate the comparison of model results in different parts of the world.

## Abstract (summary)

**Table:** 

Measurement(s)	surface soil • land • weather • evapotranspiration
Technology Type(s)	digital curation
Factor Type(s)	year
Sample Characteristic - Environment	soil environment • climate system
Sample Characteristic - Location	Earth (planet)

Machine-accessible metadata file describing the reported data: 10.6084/m9.figshare.10043090

## Background and Summary

Soil and Water Assessment Tool (SWAT)^1^ is a comprehensive hydrological model for watershed simulation. SWAT is a continuous-time, semi-distributed, and process-based model, which includes coupled upland and river processes. The land phase of SWAT includes hydrology, soil erosion, crop growth, nutrient cycling, algae transport, pesticide fate and transport, crop management, water transfer, snowfall and snowmelt, and soil temperature. The routing phase in the channels and rivers include processes such as flood routing, sediment routing, nutrient routing, pesticide routing, and routing in the reservoirs. The model is now being upgraded and restructured to SWAT+, and also coupled to glacier melt, heavy metal fate and transport, and other watershed-related processes.

SWAT is the most widely used hydrological and water quality model in the world^2^. SWAT models are used in a variety of applications, including quantification of water resources availability^3–10^, the impact of climate and landuse changes^11–16^, soil erosion^17–19^, water quality^4,20–25^, and ecosystem services^26–28^. SWAT also contains a modified version of the Environmental Policy Integrated Climate (EPIC) model^29,30^ for crop yield simulation^8,12,31^. In total, more than 4,500 ISI publications can be found using SWAT on various watershed-related issues and ecosystem services around the world, which is by far the most extensive collection of such literature in the world with an average of 550 peer-reviewed publications per year in the last 4 years (data gathered from the Web of Science, October 2019).

Lack of data in many parts of the world is a severe impediment to hydrologic modeling. At the same time, much data generated on the global and local scales is also posing a modeling problem creating an additional source of uncertainty. Previous works have shown that the use of different databases for the same region leads to different model outputs and, consequently, different water resources estimates and different estimates of ecosystem variables^4,32^. Next to model uncertainty, we have previously used the term *conditionality*^4,33,34^ to describe another constraint to a so-called calibrated model. All calibrated model parameters are uniquely conditioned on model assumptions, model structure, input data, as well as calibration data, calibration routines, and objective function definition. A calibration program, SWAT-CUP (SWAT Calibration and Uncertainty Procedures)^25,35^, was developed for the calibration of SWAT models. SWAT-CUP provides five different calibration routines and the option of choosing between 11 different objective functions. We have previously shown that the choice of different routines and objective functions lead to different parameters while producing equally acceptable calibration results^36,37^. It would be desirable to always obtain unconditional model parameters independent of calibration procedures and objective functions. For this reason, in the new version of the program, we have provided an option for multi-objective calibration, which provides an option of choosing any combination of the objective functions.

Furthermore, data processing and formatting of data for different applications are highly time-consuming and prone to errors, resulting in much of the research time to be spent on data preparation instead of modeling application and analyses. For this reason, we have put together global soil, landuse, and historical and future weather databases for use in SWAT and other similar watershed models (Table 1) as described in the next section. The collection of these data provides a valuable resource for modeling, especially in regions of data scarcity.

**Table 1:** Sources and resolutions of databases available at the Pangaea and www.2w2e.com website.

Data Type	Resolution	Source
Soil	5 km	- FAO/UNESCO global soil map
	(1995)	http://www.fao.org/soils-portal/soil-survey/soil-maps-and-databases/faounesco-soil-map-of-the-world/en/
Soil	1 km	- Harmonized World Soil Database v 1.21
	(1995)	http://webarchive.iiasa.ac.at/Research/LUC/External-World-soil-database/HTML/index.html?sb=1
Landuse	0.3 km	- GlobCover European Space Agency
	(2004–2006)	http://due.esrin.esa.int/page_globcover.php
		http://www.fao.org/land-water/land/land-governance/land-resources-planning-toolbox/category/details/en/c/1036356/
Landuse	1 km	- Global Land Cover Characterization, USGS
	(1992–1993)	http://landcover.usgs.gov/glcc/
		https://archive.usgs.gov/archive/sites/landcover.usgs.gov/globallandcover.html
Climate	0.5°	- Climate Research Unit (CRU)
	(1970–2005)	https://crudata.uea.ac.uk/cru/data/hrg/
Actual Evapo-transpiration	0.5°	- Remote sensing global monthly Actual Evapotranspiration dataset (NASA-MODIS
	(1983–2006)	http://files.ntsg.umt.edu/data/ET_global_monthly_ORIG/Global_HalfDegResolution/
GCM1	0.5°	GFDL-ESM2M, daily, RCP (2.6, 4.5, 6.0, 8.5), NOAA/Geophysical Fluid Dynamics Laboratory
	(1960–2099)	https://www.isimip.org/gettingstarted/details/51/
GCM2	0.5°	HadGEM2-ES, daily, RCP (2.6, 4.5, 6.0, 8.5), Met Office Hadley Center
	(1960–2099)	https://portal.enes.org/models/earthsystem-models/metoffice-hadley-centre/hadgem2-es
GCM3	0.5°	IPSL-CM5A-LR, daily, RCP (2.6, 4.5, 6.0, 8.5), L’Institut Pierre-Simon Laplace
	(1960–2099)	https://cmc.ipsl.fr/international-projects/cmip5/
GCM4	0.5°	MIROC, daily, RCP (2.6, 4.5, 6.0, 8.5), AORI, NIES and JAMSTEC
	(1960–2099)	https://translate.google.com/translate?hl=en&sl=ja&u=http://ccsr.aori.u-tokyo.ac.jp/project.html&prev=search
GCM5	0.5°	NorESM1-M, daily, RCP (2.6, 4.5, 6.0, 8.5), Norwegian Climate Center
	(1960–2099)	https://portal.enes.org/models/earthsystem-models/ncc/noresm

## Methods

### Soil maps of the world

#### FAO/UNESCO soil map of the world

There is a general lack of reliable soil information for many parts of the world, which has significantly disadvantaged evaluation of soil erosion, land degradation, environmental impact studies, and sustainable land management programs. Two highly-used global soil maps are the FAO/UNESCO Soil Map of the World and Harmonized World Soil Database (HWSD_v121). Both maps provide a limited description of parameters, which are not directly useful for hydrologic models. We have, therefore, used pedotransfer functions developed from soils around the world to create the needed parameters such as hydraulic conductivity, available water capacity, and bulk density. Pedotransfer functions “translate data we have into data we need”^38^. These functions estimate parameters that are difficult to measure using easily measured soil properties such as texture, color, and structure, that are routinely recorded by soil surveyors^39^.

**Fig. 1:**
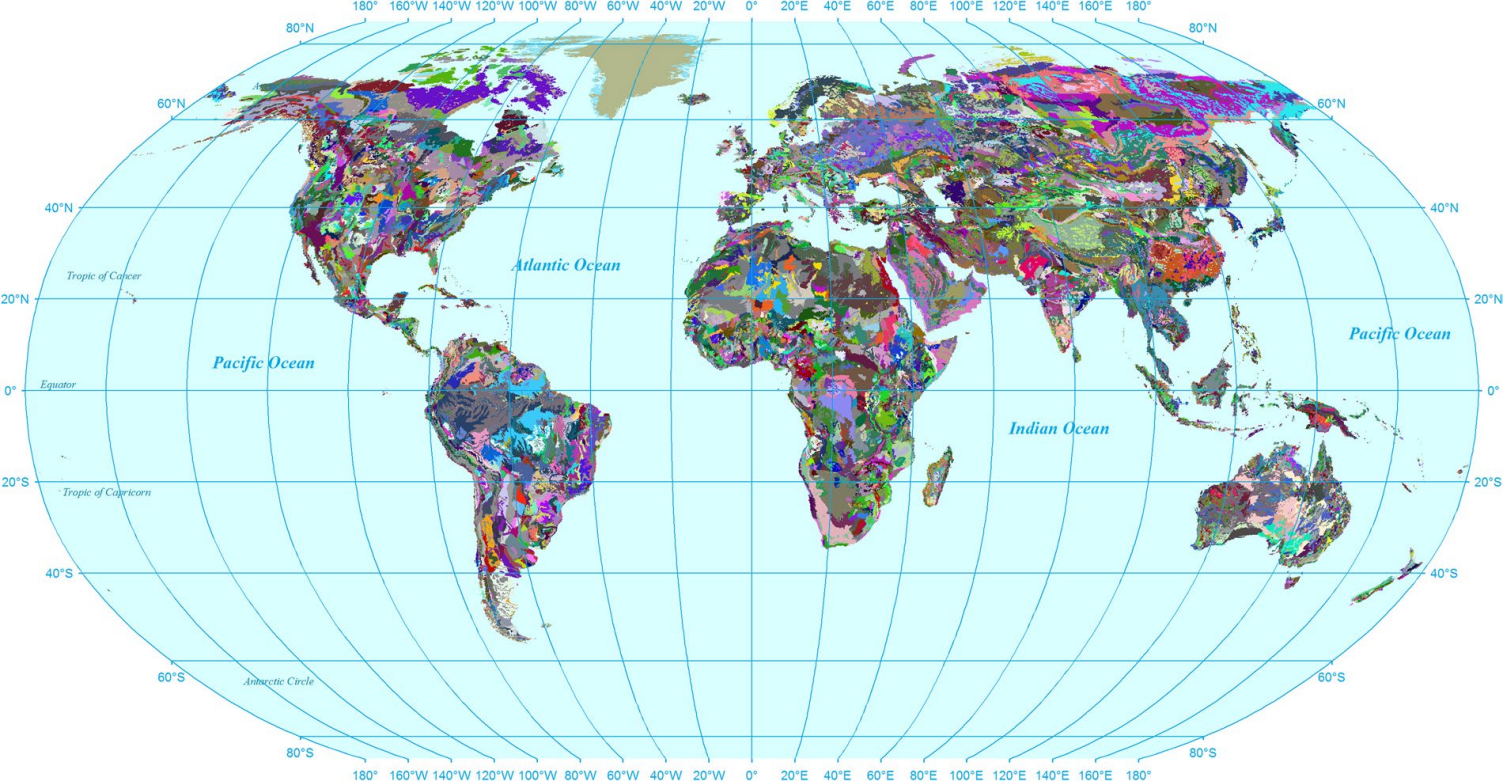
Unique soil units in FAO/UNESCO Soil Map of the World.

The FAO/UNESCO soil map of the world was prepared using the topographic map series of the American Geographical Society of New York at a nominal scale of 1:5,000,000 consisting of a 30 cm topsoil layer, and a 70 cm subsoil layer (Fig. 1). Associated files, which we produced, include “Lookup_Soil_FAO-UNESCO.txt,” which contains the correspondence between soil map and soil database, and the SWAT’s *usersoil* table in the main SWAT database “SWAT2012.mdb”.

Initially, in 2004, the first author created the soil database for the FAO/UNESCO 1995 soil map for quantification of water availability and quality in Africa^9,10^. The soil names were created as a concatenation of the FAO mapping unit (e.g., Af14-3C) and FAO Soil-ID (e.g., 1) to give Af14-3C-1. Soil hydrologic groups were determined according to SWAT Manual^40^ based on the criteria in Supplementary Table S1. The fraction of anions exclusion (ANION_EXCL) was set to 0.5 according to the SWAT Manual^40^. The potential or maximum crack volume of the soil profile (SOL_CRK) expressed as a fraction of the total soil volume was set to zero as there was no information available to evaluate this parameter. Other soil properties have initially been calculated^9,10^ using the program ROSETTA^41^. In the current study, we have updated this database using a large number of pedotransfer functions, as described below.

#### Harmonized world soil database (HWSD)

The Food and Agriculture Organization of the United Nations (FAO) and the International Institute for Applied Systems Analysis (IIASA) combined the available regional and national soil information with the data already contained within the 1:5,000,000 scale FAO-UNESCO map, into a new comprehensive Harmonized World Soil Database (HWSD_v121). This map has a resolution of about 1 km (30 arc seconds) and consists of a 30-cm topsoil layer, and a 70-cm subsoil layer (Supplementary Fig. S1).

The soil variables provided in the Harmonized World Soil Database^42^ and FAO/UNESCO Soil Map of the World included soil texture (%sand, %silt, %clay), organic carbon, pH, and electrical conductivity (EC). However, from a hydrological point of view, we require parameters such as bulk density, water storage capacity, and hydraulic conductivity for different soil layers, which we used pedotransfer functions to estimate. We estimated soil bulk density (Table 2), soil available water capacity (Table 3), soil hydraulic conductivity (Table 4), soil erodibility factor for universal soil loss equation (USLE) (Table 5), and moist soil albedo (Table 6). The used pedotransfer functions are based on the soils from around the world; hence, providing parameters that are more universally applicable. The above variables were calculated for all soil records in the two soil maps.

**Table 2:** Soil Bulk Density (ρ_b_) pedotransfer function (g cm^−3^). OC = %organic carbon, C = %clay, T = %silt, S = %sand.

Bulk Density Pedotransfer Function	Reference
ρ_b_ = 100/[1.72*OC/0.224 + (100 − 1.72*OC)/1.27]	Adams^65^
ρ_b_ = 1.66 − 0.308*OC^0.5	Alexander^66^
ρ_b_ = 1.72 − 0.294*OC^0.5	Alexander^66^
ρ_b_ = exp[−2.31 − 1.079*ln(1.72*OC/100) − 0.113*(ln(1.72*OC/100))^2]	Federer^67^
ρ_b_ = exp[− 2.39 − 1.316*ln(1.72*OC/100) − 0.167*(ln(1.72*OC/100))^2]	Huntington *et al*.^68^
ρ_b_ = exp[0.263 − 0.147*ln(OC) − 0.103*(ln(OC)^2	Huntington *et al*.^68^
ρ_b_ = 1.51 − 0.113*OC	Manrique and Jones^69^
ρ_b_ = 1.66 − 0.318*OC^0.5	Manrique and Jones^69^
ρ_b_ = 0.111*1.450/[1.450*(1.72*OC/100) + 0.111*(1 − 1.72*OC/100)]	Federer *et al*.^70^
ρ_b_ = 1.524 − 0.0046*C − 0.051*OC − 0.0045*pH + 0.001*S	Bernoux *et al*.^71^
ρ_b_ = 1.398 − 0.042*OC − 0.0047*C	Bernoux *et al*.^71^
ρ_b_ = 1.578 − 0.054*OC − 0.006*T − 0.004*C	Tomasella and Hodnett^72^
ρb = 1.70398 − 0.00313*S + 0.00261*C − 0.11245*OC	Leonavičiute^73^
ρb = 1.07256 + 0.032732*ln(S) + 0.038753*ln(C) + 0.078886*ln(S) − 0.054309*ln(OC)	Leonavičiute^73^
ρ_b_ = 0.244*1.640/[1.640*1.72*OC/100 + 0.244*(1 − 1.72*OC/100)]	Post and Kwon^74^
ρ_b_ = exp(0.313 − 0.191*OC + 0.02102*C − 0.000476*C^2 − 0.00432*T)	Kaur *et al*.^75^
ρ_b_ = 0.120*1.400/[1.4*1.72*OC/100 + 0.120*(1 − 1.72*OC/100)]	Tremblay *et al*.^76^
ρ_b_ = exp(−1.81 − 0.892*ln(1.72*OC/100) − 0.092* (ln(1.72*OC/100))^2)	Prevost^77^
ρ_b_ = 0.159*1.561/[1.561*(1.72*OC/100) + 0.159*(1 − (1.72*OC/100)]	Prevost^77^
ρ_b_ = 1.5688 − 0.0005*C − 0.0090*OC	Benites *et al*.^78^
ρ_b_ = −1.977 + 4.105*(1.72*OC/100) − 1.229*ln(1.72*OC/100) − 0.103*(ln(1.72*OC/100))^2	Perie and Ouimet^79^
ρ_b_ = 0.111*1.767/[1.767*1.72*OC/100 + 0.111*(1 − 1.72*OC/100)]	Perie and Ouimet^79^
ρ_b_ = exp(0.5379 − 0.0653*(10*1.72*OC)^0.5	Han *et al*.^80^
ρ_b_ = 1.02 − 0.156*ln(1.72*OC)	Hong *et al*.^81^
ρ_b_ = 0.071 + 1.322*exp(−0.0715*OC)	Hossain *et al*.^82^

**Table 3:** Available Water Capacity, AWC( = θ_33_–θ_1500_) (cm cm^−1^) pedotransfer functions. θ_33_ = soil water content at field capacity, θ_1500_ = soil water content at wilting point, C = %clay, ρ_b_ = bulk density (g cm^−3^), T = %silt, OC = %organic carbon, S = %sand.

Available Water Capacity Pedotransfer Function	Source
θ_33_ = 0.1183 + 0.0096*C − 0.00008*C^2	Petersen *et al*.^83^
θ_1500_ = 0.0174 + 0.0076*C − 0.00005*C^2	
θ_33_ = 0.2081 + 0.0045*C + 0.0013*T − 0.0595*ρ_b_	Hall *et al*.^84^
θ_1500_ = 0.0148 + 0.0084*C − 0.000055*C^2	
θ_33_ = 0.003075*S + 0.005886*T + 0.008039*C + 0.001284*OC − 0.1434*ρ_b_	Gupta & Larson^85^
θ_1500_ = 0.000059*S + 0.001142*T + 0.005766*C + 0.001326*OC + 0.02671*ρ_b_	
θ_33_ = 0.2576 − 0.002*S + 0.0036*C + 0.0299*OC	Rawls *et al*.^86^
θ_1500_ = 0.0260 + 0.005*C + 0.0158*OC	
θ_33_ = 0.3486 − 0.0018*S + 0.0039*C + 0.0228*OC − 0.0738*ρ_b_	Rawls *et al*.^87^
θ_1500_ = 0.0854 − 0.0004*S + 0.0044*C + 0.0122*OC − 0.0182*ρ_b_	
θ_33_ = 0.3862 − 0.0000479*S − 0.000019*(S/T)^2	Rajkai & Varallyay^88^
θ_1500_ = 0.0139 + 0.0036*C + 0.006508*OC^2	
θ_33_ = 0.01*ρ_b_*(2.65 + 1.105*C − 0.01896*C^2 + 0.0001678*C^3 + 15.12*ρ_b_ − 6.745*ρ_b_^2 − 0.1975*C*ρ_b_)	Canarache^89^
θ_1500_ = 0.01*ρ_b_*(0.2805*C + 0.0009615*C^2)	
AWC = 0.000976*C + 0.001875*T + 0.004694*OC	Batjes^90^
AWC = 0.001082*C + 0.001898*T + 0.007705*OC	Batjes^90^
θ_33_ = 0.04046 + 0.00426*T + 0.00404*C	Tomasella & Hodnett^72^
θ_1500_ = 0.0091 + 0.00150*T + 0.00396*C	
x = −0.837531 + 0.430183*OC	Rawls *et al*.^91^
y = −1.40744 + 0.0661969*C	
z = −1.51866 + 0.0393284*S	
θ_33_ = 0.297528 + 0.103544*(0.0461615 + 0.290955*x − 0.0496845*x^2 + 0.00704802*x^3 + 0.269101*y − 0.176528*x*y + 0.0543138*x^2*y + 0.1982*y^2–0.060699*y^3–0.320249*z − 0.0111693*x^2*z + 0.14104*y*z + 0.0657345*x*y*z − 0.102026*y^2*z − 0.04012*z^2 + 0.160838*x*z^2–0.121392*y*z^2–0.0616676*z^3)	
θ_1500_ = 0.142568 + 0.0736318*(0.06865 + 0.108713*x − 0.0157225*x^2–0.017059*y^2 + 0.00102805*x^3 + 0.886569*y − 0.223581*x*y + 0.0126379*x^2*y + 0.013526*x*y^2–0.0334434*y^3–0.0535182*z − 0.0354271*x*z − 0.00261313*x^2*z − 0.154563*y*z − 0.0160219*x*y*z − 0.0400606*y^2*z − 0.104875*z^2 + 0.0159857*x*z^2–0.0671656*y*z^2–0.0260699*z^3)	
β = −0.00251*S + 0.00195*C + 0.0064*OC + 0.000035*S*OC − 0.00016*C*OC + 0.0000452*S*C + 0.299	Saxton and Rawls^92^
γ = −0.00024*S + 0.00487*C + 0.0035*OC + 0.00029*S*OC − 0.0000756*C*OC + 0.0000068*S*OC + 0.031	
θ_33_ = β + (1.283*β^2–0.374*β − 0.015)	
θ_1500_ = γ + (0.14*γ − 0.02)	
θ_33_ = 0.0055*(C + T) − 0.0013*S*ρ_b_ + 0.1288	Aina & Periaswamy^93^
θ_1500_ = 0.0031*C + 0.0213	
θ_33_ = 0.3697–0.0035*S	Dijerman^94^
θ_1500_ = 0.0074 + 0.0039*C	
θ_33_ = [0.0029*(C + T) + 0.0993] *ρ_b_	Arruda *et al*.^95^
θ_1500_ = [0.0027*(C + T) + 0.0107]*ρ_b_

**Table 4:** Soil Hydraulic Conductivity (cm day^−1^) pedotransfer functions. θ_33_ = soil water content at field capacity, θ_1500_ = soil water content at wilting point, C = %clay, ρ_b_ = bulk density (g cm^−3^), T = %silt, OC = %organic carbon, S = %sand, topsoil = an ordinal variable having the value of 1 for (depth 0–30 cm) or 0 (depth > 30 cm).

Hydraulic Conductivity Pedotransfer Function	Source
K_s_ = 60.96*10^(−0.884 + 0.0153*S)	Cosby *et al*.^96^
K_s_ = 60.96*10^(−0.6 + 0.0126*S − 0.0064*C)	Cosby *et al*.^96^
K_s_ = 24.0*exp(12.012–0.0755*S + α)	Saxton *et al*.^97^
α = (−3.895 + 0.03671*S − 0.1103*C + 0.00087546*C^2)/θ_s_	
θ_s_ = 0.332–0.0007251*S + 0.1276*log(C)	
Ks = 339.0*(1.3/ρ_b_)^(1.3* β)*exp(−0.0688*C − 0.0363*T − 0.025)	Campbell and Shiozawa^98^
γ = exp{0.01*[ln(1.025)*S + ln(0.026)*T + ln(0.001)*C]}	
µ = exp{0.01*[ln(1.025)]^2*S + [ln(0.026)]^2*T + [ln(0.001)]^2*C]−[ln(γ)]^2}^0.5	
β = [γ^ − 0.5 + 0.2*µ]^ − 1	
K_S_ = 4632(θ_s_–θ_33_)^(3 − λ)	Saxton and Rawls^92^
θ_s_ = θ_33_–0.064–0.00097*S + 1.636(0.00278*S + 0.00034*C + 0.0128*OC − 0.000104*S*OC − 0.000157*C*OC − 0.0000584*S*C + 0.078)	
λ = [ln(θ_33_) − ln(θ_1500_)]/[ln(1500) − ln(33)]	
θ_33_ = β + (1.283*β^2–0.374*β − 0.015)	
β = −0.00251*S + 0.00195*C + 0.0064*OC + 0.000035*S*OC − 0.00016*C*OC + 0.0000452*S*C + 0.299	
θ_1500_ = γ + (0.14*γ − 0.02)	
γ = 0.00024*S + 0.00487*C + 0.0035*OC + 0.00029*S*OC − 0.0000756*C*OC + 0.0000068*S*OC + 0.031	
θ_s_ = 1 − ρ_b_/2.65	Rawls and Brakensiek^99^
K_S_ = 24*exp(α)	
α = 19.52348*θ_s_ − 8.96847–0.028212*C + 0.00018107*(S^2) − 0.0094125*(C^2) − 8.395215*(θ_s_^2) + 0.077718*S*θ_s_ − 0.00298*(S^2)*(θ_s_^2) − 0.019492*(C^2)*(θ_s_^2) + 0.0000173*(S^2)*C + 0.02733*(C^2)*θ_s_ + 0.001434*(S^2)*θ_s_ − 0.0000035*S*(C^2)	
K_S_ = exp(α)	Woesten *et al*.^100^
α = 7.755 + 0.0352*T + 0.93(topsoil) − 0.967*(ρ_b_^2) − 0.000484*C^2–0.000322*(T^2) + 0.001/T − 0.129/OC − 0.643*ln(T) − 0.01398*ρ_b_*C − 0.0973*ρ_b_*OC + 0.02986(topsoil)*C − 0.03305*(topsoil)*T	
K_S_ = exp(α)	Weynants *et al*.^101^
α = 1.9582 + 0.0308*S – 0.6142*ρ_b_ – 0.01566*OC

**Table 5:** Soil erodibility factor (cm day^−1^) pedotransfer function. S = %sand, T = %silt, C = %clay, OC = %organic carbon.

Soil Erodibility Pedotransfer Function	Source
K_USLE_ = E_S_*E_C-T_*E_OC_*E_HS_	Williams^30^
Where:	
E_s_ = 0.2 + 0.3*exp[−0.256*S*(1 − T/100)]	
E_C-T_ = [T/(C + T)]^0.3	
E_OC_ = 1−(0.25*OC/(OC + exp(0.72 − 2.95*OC)]	
E_HS_ = 1−{0.7*(1 − S/100)/[(1 − S/100) + exp(−5.51 + 22.9*(1 − S/100)]}

**Table 6:** Moist Soil Albedo based on the water content at field capacity (θ_33_).

Albedo Pedotransfer Function	Source
Albedo = 0.1807 + 0.1019*exp(−3.53*θ_33_)	Wang *et al*.^102^
Albedo = 0.15 + 0.31*exp(−12.7*θ_33_)	Gascoin *et al*.^103^
Albedo = 0.26 + 0.1068*exp(−4.9*θ_33_)	Sugathan *et al*.^104^

Furthermore, to account for parameter uncertainty, the soils were sorted by their textural classes based on USDA classification^42^ that included Clay, Clay-loam, Heavy-clay, Loam, Loamy-sand, Sand, Sandy-clay, Sandy-clay-loam, Sandy-loam, Slit-loam, Silty-clay, and Silty-clay-loam. For each textural class, we pooled the estimates of various pedotransfer functions from both FAO_UNESCO and HWSD databases and calculated their cumulative probability distributions from which we obtained parameter values at the 5%, 50%, and 95% probability levels. Values for bulk density are shown in Table 7 as an example, while other parameters are given in Supplementary Tables S2–S6. An example calculation of the 95 percent prediction uncertainty (95PPU) is shown in Supplementary Fig. S2 for the hydraulic conductivity of topsoil sandy loam. The 95PPU parameter range sets a physically meaningful limit on the parameters for different soil textural classes and is instrumental in constraining the respective parameters in model calibration. These ranges can, of course, be modified by the user as needed.

**Table 7:** Average and uncertainty estimates of bulk density for top and subsoil based on the textural classes. The numbers in the brackets are the number of samples.

Topsoil Bulk density (g cm^−3^)	5% prob. Level	50% prob. Level	95% prob. Level	Subsoil Bulk density (g cm^−3^)	5% prob. Level	50% prob. Level	95% prob. Level
Clay (2324)	0.80	1.19	1.58	Clay (2221)	1.19	1.34	1.49
Clay-loam (3034)	1.03	1.30	1.57	Clay-loam (4936)	1.19	1.37	1.55
Heavy-clay (284)	1.04	1.21	1.38	Heavy-clay 548)	1.21	1.32	1.42
Loam (6612)	0.98	1.26	1.54	Loam (5150)	1.08	1.34	1.61
Loamy-sand (1171)	1.11	1.30	1.49	Loamy-sand (1072)	1.31	1.40	1.50
Sand (918)	1.33	1.41	1.49	Sand (793)	1.38	1.41	1.44
Sandy-clay (136)	1.10	1.27	1.44	Sandy-clay (461)	1.15	1.39	1.62
Sandy-clay-loam (2463)	1.19	1.34	1.49	Sandy-clay-loam (2518)	0.97	1.36	1.74
Sandy-loam (3040)	1.08	1.32	1.57	Sandy-loam (2533)	1.14	1.37	1.60
Silt-loam (864)	0.79	1.19	1.60	Silt-loam (608)	0.74	1.26	1.77
Silty-clay (120)	0.88	1.17	1.47	Silty-clay (142)	1.04	1.32	1.59
Silty-cla-loam (95)	0.95	1.21	1.47	Silty-cla-loam (89)	1.19	1.36	1.53

In the pre-processing of HWSD database, similar to FAO/UNESCO, we modified the data where necessary by replacing zero values of %sand, %silt, and %clay by 1, and making sure that their summation equals 100%. Also, after applying various pedotransfer functions, we replaced the negative or unreasonable values with the overall averages to avoid model-generated errors. Finally, we should point out that the soil parameters in both databases must still be calibrated for a specific location.

### Landcover maps of the world

#### Global land cover characterization (GLCC)

The GLCC from USGS is a landuse and land cover classification dataset based primarily on the unsupervised classification of the 1-km AVHRR (Advanced Very High-Resolution Radiometer) 10-day NDVI (Normalized Difference Vegetation Index) composites (Supplementary Fig. S3). The AVHRR source imagery dates from April 1992 through March 1993. The GLCC map contains 24 land cover types. We made the correspondence between the GLCC map units and SWAT’s (*crop*) database in Supplementary Table S7 based on the description of the land covers provided by the maps and the SWAT landuse definitions.

#### Global landuse GlobCover

The GlobCover is a European Space Agency initiative to develop global composites and land cover maps using observations from the 300-m MERIS sensor onboard the ENVISAT satellite mission (Soolementary Fig. S4). The GlobCover map covers the period of December 2004 to June 2006 and is derived by automatic and regionally-tuned classification of a MERIS full resolution surface reflectance time series. The GlobCover map contains 23 land cover types. We made correspondence between the GlobCover units and SWAT’s (*crop*) database in Supplementary Table S8 based on the description of the land covers provided by the maps and the SWAT landuse definitions.

The databases for the above two global landuse maps are supported by the table (*crop*) in the SWAT2012.mdb database and the lookup tables “Lookup_Landuse_GlobCover.txt” and Lookup_Landuse_USGS.txt. However, similar to the soil parameters, landuse parameters must be calibrated for a given location.

### Historical weather data

The historical (1970–2005) reanalysis temperature and precipitation data from the Research Unit East Anglia (CRU TS 3.1)^43^ were reformatted from NetCDF into SWAT-readable text files. The database is daily and has a resolution of 0.5° and covers the entire globe in 67,420 files.

### Future weather data

We provide five global climate models (GCM), each with four carbon evolution scenarios supported by ISI-MIP5 (Inter-Sectoral Impact Model Intercomparison Project)^44^. These daily data cover the period of 1950–2099 and have a resolution of 0.5°. Similar to CRU, they have been reformatted from NetCDF into SWAT-formatted text files.

The five GCM models include HadGEM2-ES, IPSL-CM5A-LR, MIROC-ESM-CHEM, GFDL-ESM2M, and NorESM1-M (Table 1) with Representative Concentration Pathway (RCP) scenarios (RCP2.6, RCP4.5, RCP6.0, and RCP8.5)^45^. The 0.5° grid WATCH Forcing Data^46^ for the period of January 1, 1960, to December 31, 1999 (the reference period) was used as observation data to downscale the five GCMs^44^. WATCH is a combination of the ERA-40 daily data, the 40-year reanalysis of the European Centre for Medium-Range Weather Forecasts, and the Climate Research Unit TS2.1 dataset (CRU)^43^. The WATCH Forcing Data data combines the daily statistics of ERA-40 with the monthly mean characteristics of CRU and Global Precipitation Climatology Centre (GPCC) datasets and represents a complete gridded observational dataset for bias correction of global climate data^44^.

The historical and future data can be downloaded for any given geographic location from www.2w2e.com using the template illustrated in Supplementary Fig. S5. The Climate Change Toolkit (CCT) program^47^ is linked to the above databases and can be used for bias correction if local data is available. CCT uses additive correction for temperature and a multiplicative correction factor for precipitation. The program can also be used for extreme climate analysis^48^.

### Global actual evapotranspiration data

Actual evapotranspiration (AET) from the earth’s land surface is collected by NASA using satellite data from 1982 to 2003^49,50^ (Supplementary Fig. S6). The algorithm calculates canopy transpiration and soil evaporation using a modified Penman-Monteith approach with biome-specific canopy conductance determined from the normalized difference vegetation index (NDVI). Priestley-Taylor approach was used to quantify open water evaporation. The observations from 34 flux network (FEUXNET) tower sites^51^ were used to parameterize an NDVI-based canopy conductance model to validate the global ET al.gorithm using measurements from 48 additional, independent flux towers^49,50^.

**Fig. 2:**
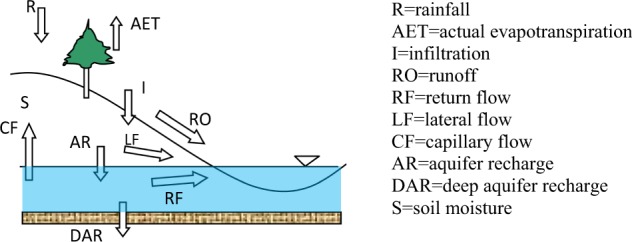
Schematic illustration of the conceptual water balance model in SWAT.

AET has been used before to calibrate SWAT when other observed data is not available^52^. It is crucial to have a measure of AET when calibrating a SWAT model with river discharge data. Using river discharge alone, we can confidently estimate runoff and infiltration. However, components of the infiltrated water cannot be estimated with any degree of confidence. These components include soil moisture (S), aquifer recharge (AR), and actual evapotranspiration (AET) (Fig. 2). Using an estimate of AET in calibration can significantly increase our confidence in the other components of infiltrating water.

To use the provided MODIS–NASA data for calibration in SWAT-CUP, users, should overlay the MODIS-AET grids with the subbasin map of their ArcSWAT/QSWAT project and average the AET grid points inside each subbasin to one single value to represent the subbasin’s AET.

## Data Records

The Global FAO/UNESCO Soil Map of the World and associated SWAT data files (Lookup Table and SWAT2012.mdb)^53^ are deposited at *Pangaea* and www.2w2e.com sites. There are 4,931 soil records in this data set.

The Harmonized World Soil map and associated SWAT data files (Lookup Table and SWAT2012.mdb)^54^ are deposited at *Pangaea* and www.2w2e.com sites. There are 16,328 soil records in this data set.

The Global Land Cover Characterization (GLCC) map from USGS and SWAT data file (Lookup Table and SWAT2012.mdb)^55^ are deposited at *Pangaea* and www.2w2e.com sites. There are 24 landcover types in this database.

The GlobCover from the European Space Agency and associated data files (Lookup Table and SWAT2012.mdb)^56^ are deposited at *Pangaea* and www.2w2e.com sites. There are 23 landcover types in this database.

The historical CRU and future GCM weather data^57^ are deposited at *Pangaea* and www.2w2e.com.

Finally, the Global Actual Evapotranspiration Data^58^ in text format is deposited at *Pangaea* and www.2w2e.com.

## Technical Validation

The global soil and landuse databases have been successfully used in many SWAT applications around the world^4,6,9,10,16,59–62^. Validation of these maps, which are based on satellite observations, are offered by ground-truth observations conducted by the map developers and also in various literature^63,64^.

There is a significant variation in the reported values of soil parameters in the literature and by various agencies. In this research, we used a large number of pedotransfer functions and soil samples from around the world to estimate the textural-based soil parameters. In Table 8 we compared our estimated values of bulk density and hydraulic conductivity with values reported by the U.S. Department of Agriculture (USDA), STRUCTx (STRUCTURAL ENGINEERING RESOURCES website, see Table 8), and other reported values. The rest of the parameters could not be found based on textural classes. As evident, there are significant variations in all estimates, especially for saturated hydraulic conductivities. For this reason, it is essential to have a range of estimates, so one can limit the values to a likely range during model calibration.

**Table 8:** Comparison of the values of bulk density and saturated hydraulic conductivity estimated in this research with values reported by different sources.

Topsoil Bulk density (g cm^−3^)	5% prob. Level	50% prob. Level	95% prob. Level	USDA^*^	STRUCTx^**^	From Articles^***^
Clay (2324)	0.80	1.21	1.58	1.4	1.33	—
Clay-loam (3034)	1.03	1.26	1.57	1.45	1.39	1.44–1.59
Heavy-clay (284)	1.04	1.30	1.38	1.3	—	—
Loam (6612)	0.98	1.41	1.54	1.5	1.43	1.28–1.60
Loamy-sand (1171)	1.11	1.27	1.49	1.6	1.43	1.33–1.82
Sand (918)	1.33	1.34	1.49	1.65	1.43	1.27–1.75
Sandy-clay (136)	1.10	1.32	1.44	1.4	1.47	1.55–1.65
Sandy-clay-loam (2463)	1.19	1.19	1.49	1.5	1.5	1.49–1.75
Sandy-loam (3040)	1.08	1.17	1.57	1.55	1.46	1.4–1.76
Silt-loam (864)	0.79	1.21	1.60	1.5	1.38	1.12–1.61
Silty-clay (120)	0.88	1.16	1.47	1.45	1.26	0.95–1.33
Silty-clay-loam (95)	0.95	1.23	1.47	1.5	1.3	0.86–1.60
*https://www.nrcs.usda.gov/wps/portal/nrcs/detail/soils/survey/office/ssr10/tr/?cid=nrcs144p2_074844						
**https://structx.com/Soil_Properties_002.html						
***Ranges from^105^ & https://hort.ifas.ufl.edu/woody/critical-value.shtml &						
**Topsoil Saturated Hydraulic Conductivity (mm hr** ^**−1**^ **)**	**5% prob. Level**	**50% prob. Level**	**95% prob. Level**	USDA*	STRUCTx**	From articles (SD)***
Clay	3	5	13	2–5	5	40 (255)
Clay-loam	6	9	13	5–15	9	13 (46)
Heavy-clay	3	3	6	2–5	—	—
Loam	8	12	22	15–51	25	58 (113)
Loamy-sand	70	101	130	151–508	562	98 (133)
Sand	111	117	176	151–508	634	330 (328)
Sandy-clay	10	15	26	2–5	8	27 (83)
Sandy-clay-loam	12	19	44	5–15	23	32 (170)
Sandy-loam	20	47	78	51–152	124	49 (183)
Silt-loam	7	10	54	15–51	26	52 (96)
Silty-clay	3	4	4	2–5	4	—
Silty-clay-loam	3	4	7	2–5	6	180 (434)
*https://www.nrcs.usda.gov/wps/portal/nrcs/detail/soils/survey/office/ssr10/tr/?cid=nrcs144p2_074846						
**https://structx.com/Soil_Properties_007.html						
***Based on García-Gutiérrez *et al*.^106^						

## Usage Notes

There are 4,931 soil records in the FAO/UNESCO database, and 16,328 records in the HWSD soil map. Both (*usersoil*) tables are in the SWAT2012.mdb database. The field (*Name*) is concatenated by using the fields SU-SYM74, SU-SYM90, MU_GLOBAL, and ISSOL as given in the original HWSD database. SU-SYM74 is the soil unit symbol according to the FAO-74 soil classification, SU-SYM90 is the soil unit symbol according to the FAO-90 soil classification, MU_GLOBAL is the Global Mapping Unit identifier, which provides the link between the GIS soil units and the attribute database, and ISSOL is a field indicating if the soil mapping unit is a soil (1) or a non-soil (0). All maps provided have the World WGS-84 Spatial Reference without any projection. The users will have to project these maps as needed before using it in the ArcSWAT or QSWAT models.

Different soil and landuse maps are provided to emphasize the fact that often more than one database is available for building and calibrating a model, and also to encourage the users to use different databases to realize the *conditionality* of their calibrated models. Calibrated model parameters are always conditioned on the input data, meaning one could obtain a different set of parameters if one had used a different set of available data. This is probably the most disappointing aspect of calibration.

A calibrated model is, therefore, always: non-unique, subjective, conditional, and subsequently limited on the scope of its use. To achieve *unconditionality*, the calibrated parameters must be integrated over all conditioning factors. Hence, we recommend using different physical inputs and multi-objective calibration procedures.

## Supplementary information

Supplementary Information.
