# Supplementary material for: Global soil, landuse, evapotranspiration, historical and future weather databases for SWAT Applications
Source: Sci Data. 2019 Nov 6;6:263. doi: 10.1038/s41597-019-0282-4 (PMC6834600; doi:10.1038/s41597-019-0282-4)
Supplement: Supplementary file 1 — Supplementary Information. [file 41597_2019_282_MOESM1_ESM.docx]

**Supplementary Material**

**Global soil, landuse, evapotranspiration, historical and future weather databases formatted for SWAT applications**

**K.C. Abbaspour^1^, S. Ashraf Vaghefi^1^, H. Yang^1^, R. Srinivasan^2^**

| Criteria | Soil Hydrologic Group | | | |
| --- | --- | --- | --- | --- |
|  | A | B | C | D |
| Mean permeability of  surface layer (mm hr^-1^) | > 254.0 | 84.0-254.0 | 8.4-84.0 | < 8.4 |

**Table S1.** Soil hydrologic group rating criteria^39^.


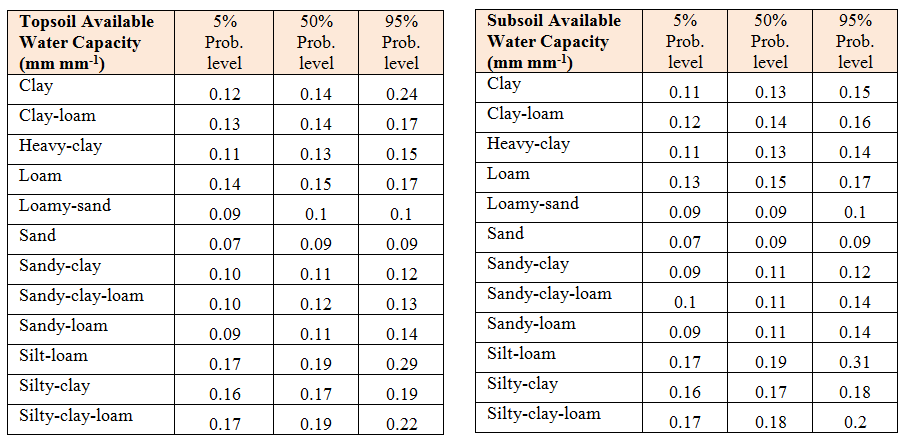


**Table S2.** Average and uncertainty estimates of available water capacity for top and subsoil based on the textural classes.


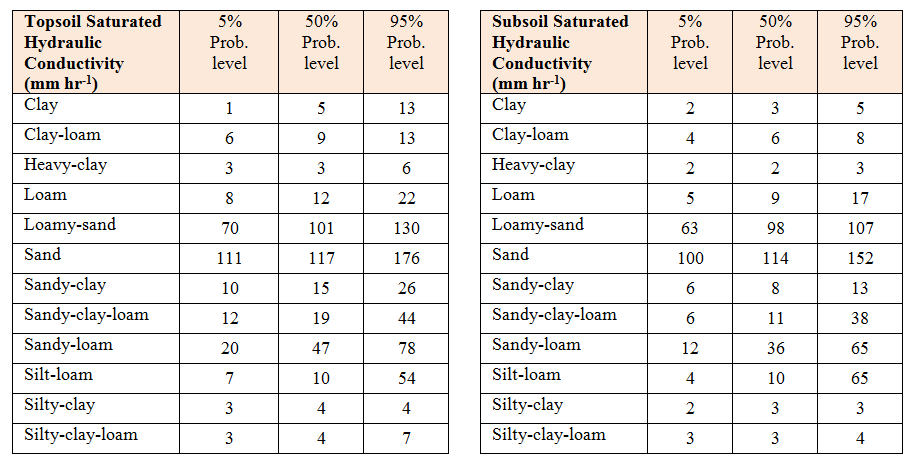


**Table S3.** Average and uncertainty estimates of saturated hydraulic conductivity for top and subsoil based on the textural classes.


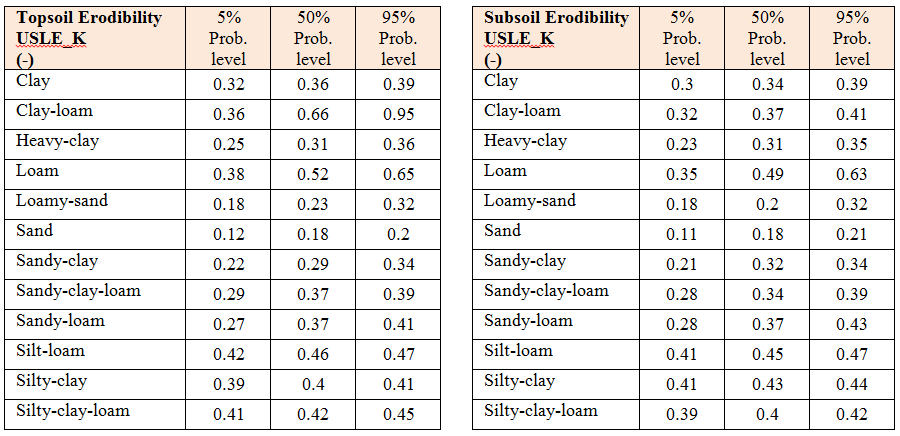


**Table S4.** Average and uncertainty estimates of soil erodibility for top and subsoil based on the textural classes.


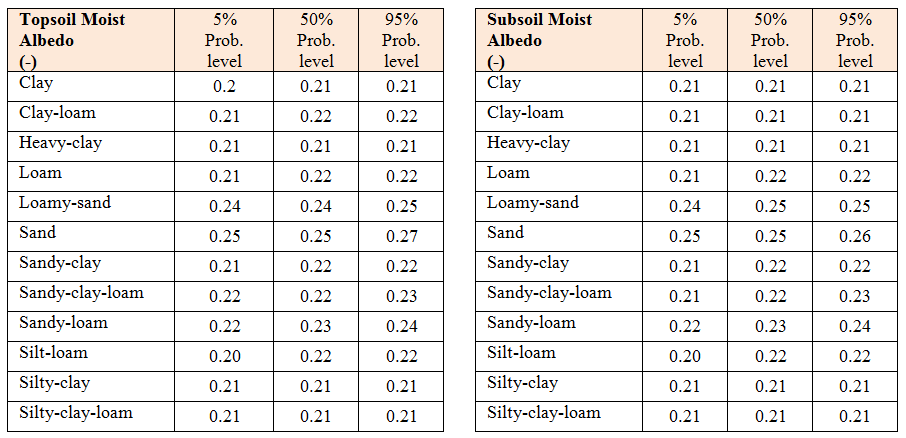


**Table S5.** Average and uncertainty estimates of moist albedo for top and subsoil based on the textural classes.


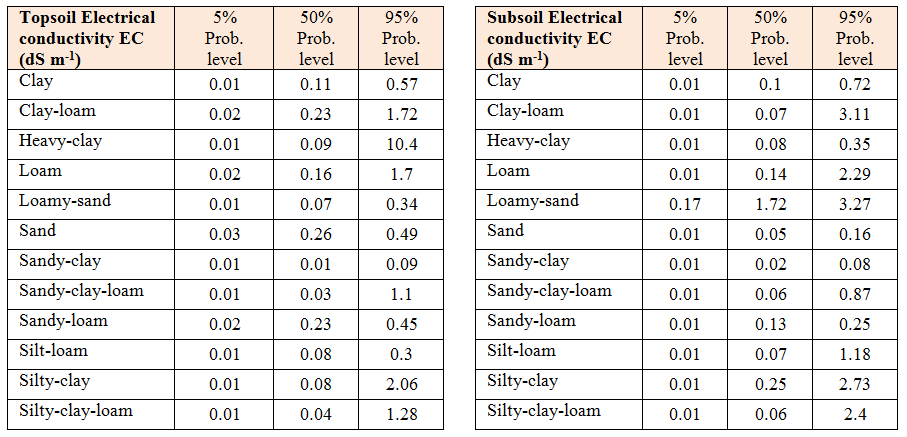


**Table S6.** Average and uncertainty estimates of electrical conductivity for top and subsoil based on the textural classes.

|  | **USGS-definition** | **Crop name in the database** | **SWAT definition** |
| --- | --- | --- | --- |
| 0 |  |  |  |
| 1 | Urban and Built-Up Land | URMD | Residential Medium density |
| 2 | Dryland Cropland and Pasture | CRDY | Avg(Ag.Land gen. and Pasture) |
| 3 | Irrigated Cropland and Pastur | CRIR | Avg(Ag.Land gen. and Pasture) |
| 4 | Mixed Dryland/Irrigated Cropl | MIXC | Agricultural Land-Generic |
| 5 | Cropland/Grassland Mosaic | CRGR | Avg(Ag.Land gen. and Range-Grasses) |
| 6 | Cropland/Woodland Mosaic | CRWO | Avg(Ag.Land.gen and Forest Mixed) |
| 7 | Grassland | GRAS | Range-Grasses |
| 8 | Shrubland | SHRB | Range-Brush |
| 9 | Mixed Shrubland/Grassland | MIGS | Avg(Range-Grass and Range-Brush) |
| 10 | Savanna | SAVA | Avg(Range-Grass and SW US Arid Range) |
| 11 | Deciduous Broadleaf Forest | FODB | Forest-Deciduous |
| 12 | Deciduous Needleleaf Forest | FODN | Forest-Deciduous |
| 13 | Evergreen Broadleaf Forest | FOEB | Forest-Evergreen |
| 14 | Evergreen Needleleaf Forest | FOEN | Forest-Evergreen |
| 15 | Mixed Forest | FOMI | Forest-Mixed |
| 16 | Water Bodies | WATB | Water |
| 17 | Herbaceous Wetland | WEHB | Wetlands-Mixed |
| 18 | Wooded Wetland | WEWO | Wetlands-Forested |
| 19 | Barren or Sparsely Vegetated | BSVG | Southwestern US (Arid) Range |
| 20 | Herbaceous Tundra | TUHB | Altai Wildrye |
| 21 | Wooded Tundra | TUWO | Avg(altai wildrye and range brush, temp reduced ) |
| 22 | Mixed Tundra | TUMI | Avg(SW US arid Range and altai wildrye, temp reduced) |
| 23 | Bare Ground Tundra | TUBG | Avg(SW US arid Range and altai wildrye, temp reduced) |
| 24 | Snow or Ice | ICES | Water |

**Table S7.** Matching of USGS Global Land Cover Characterization database to SWAT’s *crop* database.

| Value | **GlobCover definition** | **Code** | **SWAT code and landuse Definition** |
| --- | --- | --- | --- |
| 11 | Post-flooding or irrigated croplands (or aquatic) | AGIR | AGRL, Agricultural Land-Generic |
| 14 | Rainfed croplands | AGRF | AGRL, Agricultural Land-Generic |
| 20 | Mosaic cropland (50-70%) / vegetation (grassland/shrubland/forest) (20-50%) | AGMX | AGRL, Agricultural Land-Generic |
| 30 | Mosaic vegetation (grassland/shrubland/forest) (50-70%) / cropland (20-50%) | CRGR | CRGR, Cropland/Grassland Mosaic |
| 40 | Closed to open (>15%) broadleaved evergreen or semi-deciduous forest (>5m) | FRSE | FRST, Forest-Evergreen |
| 50 | Closed (>40%) broadleaved deciduous forest (>5m) | FRSD | FRSD, Forest Deciduous |
| 60 | Open (15-40%) broadleaved deciduous forest/woodland (>5m) | FRST | FRST, Forest-Mixed |
| 70 | Closed (>40%) needleleaved evergreen forest (>5m) | PINE | PINE, Pine |
| 90 | Open (15-40%) needleleaved deciduous or evergreen forest (>5m) | FRST | FRST, Forest-Mixed |
| 100 | Closed to open (>15%) mixed broadleaved and needleleaved forest (>5m) | FRST | FRST, Forest-Mixed |
| 110 | Mosaic forest or shrubland (50-70%) / grassland (20-50%) | RNG1 | RNGB, Range-Brush, modified for cold temperature and leaf area Index |
| 120 | Mosaic grassland (50-70%) / forest or shrubland (20-50%) | RNE1 | RNGE, Range-Grasses |
| 130 | Closed to open (>15%) (broadleaved or needleleaved, evergreen or deciduous) shrubland (<5m) | FRST | FRST, Forest-Mixed |
| 140 | Closed to open (>15%) herbaceous vegetation (grassland, savannas or lichens/mosses) | RYER | RYER, Russian Wildrye |
| 150 | Sparse (<15%) vegetation | RNGB | RNGB, Range-Brush |
| 160 | Closed to open (>15%) broadleaved forest regularly flooded (semi-permanently or temporarily) - Fresh or brackish water | WETF | WETF, Wetlands-Forested |
| 170 | Closed (>40%) broadleaved forest or shrubland permanently flooded - Saline or brackish water | WETF | WETF, Wetlands-Forested |
| 180 | Closed to open (>15%) grassland or woody vegetation on regularly flooded or waterlogged soil - Fresh, brackish or saline water | WETN | WETN, Wetlands-Non-Forested |
| 190 | Artificial surfaces and associated areas (Urban areas >50%) | URBN | Residential |
| 200 | Bare areas | BSVG | BSVG, Baren Or Sparsely Vegetated |
| 210 | Water bodies | WATB | WATB, Water Bodies |
| 220 | Permanent snow and ice | ICES | ICES, Snow or Ice |
| 230 | No data (burnt areas, clouds) | BARR | BARR, Barren |

**Table S8.** Conversion of Global Landuse GlobCover to SWAT’s *crop* database.


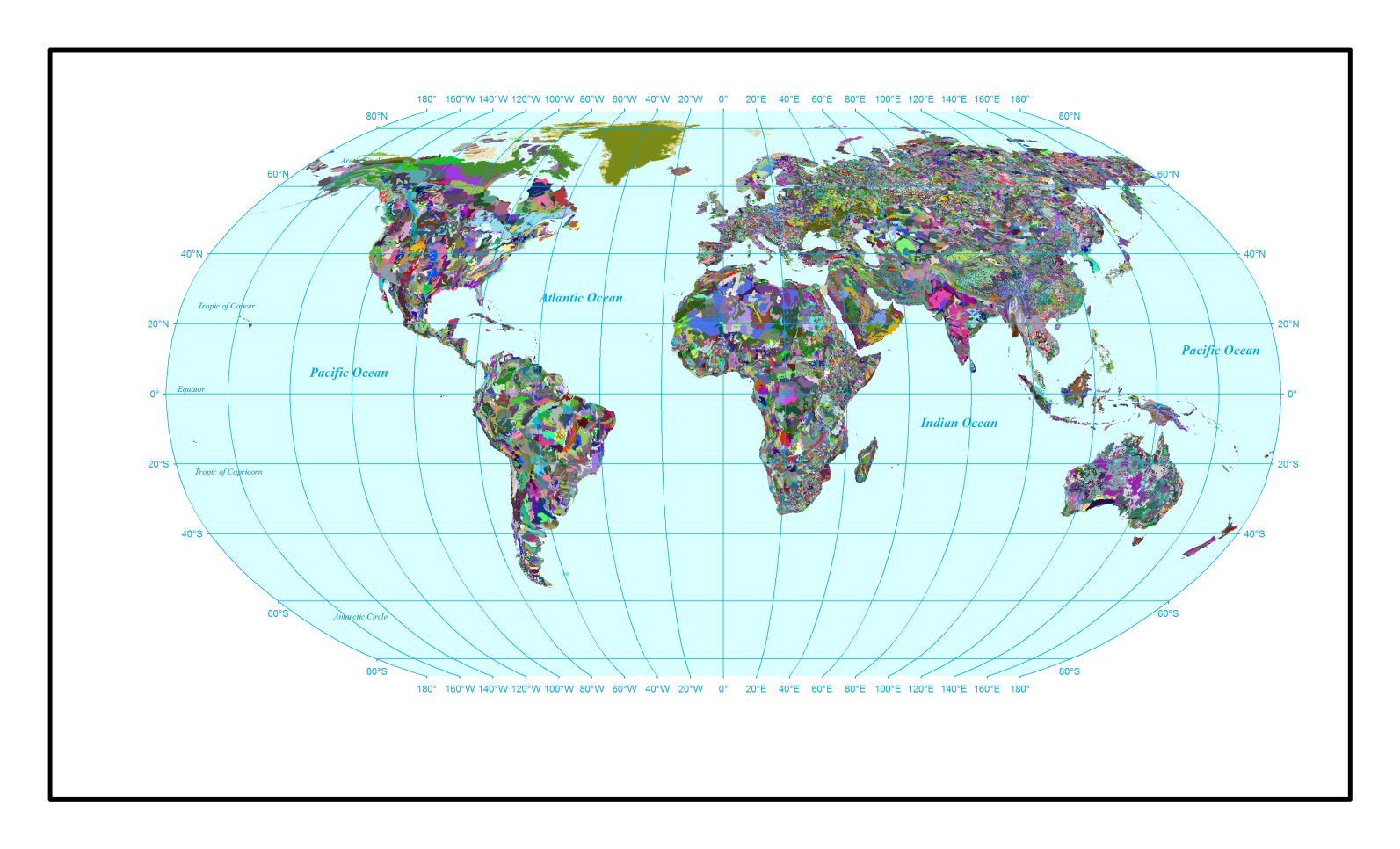


**Figure S1.** Unique soil units in the Harmonized World Soil Database (HWSD).


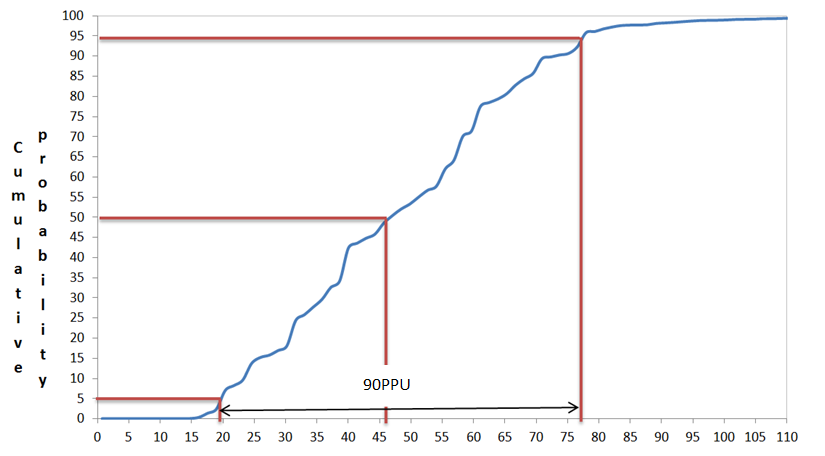


**Figure S2.** Definition of the 90% prediction uncertainty (90PPU) of a variable using the cumulative probability calculated based on many samples.


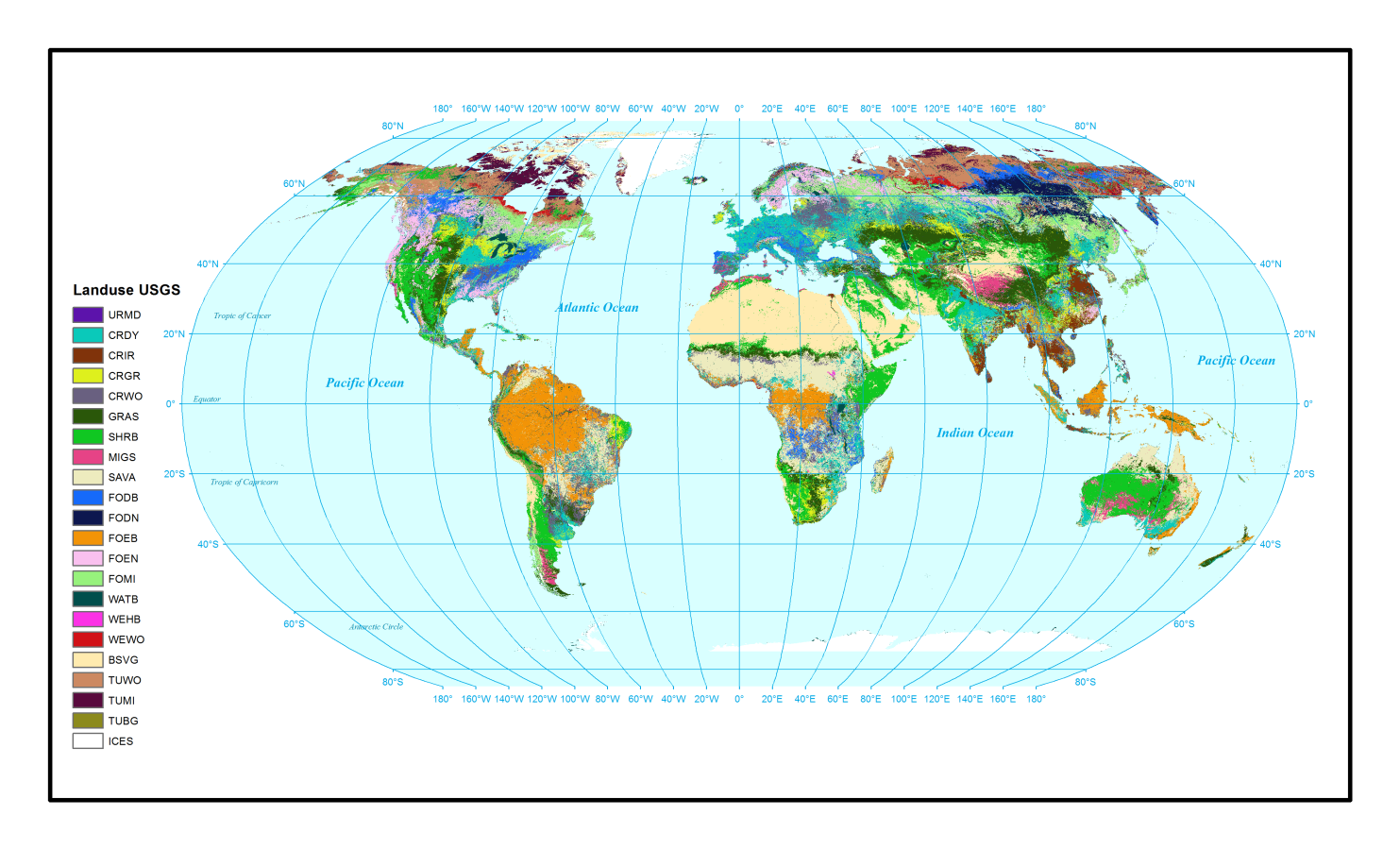


**Figure S3.** Unique landcover in the USGS Landuse/Land Cover Characterization.


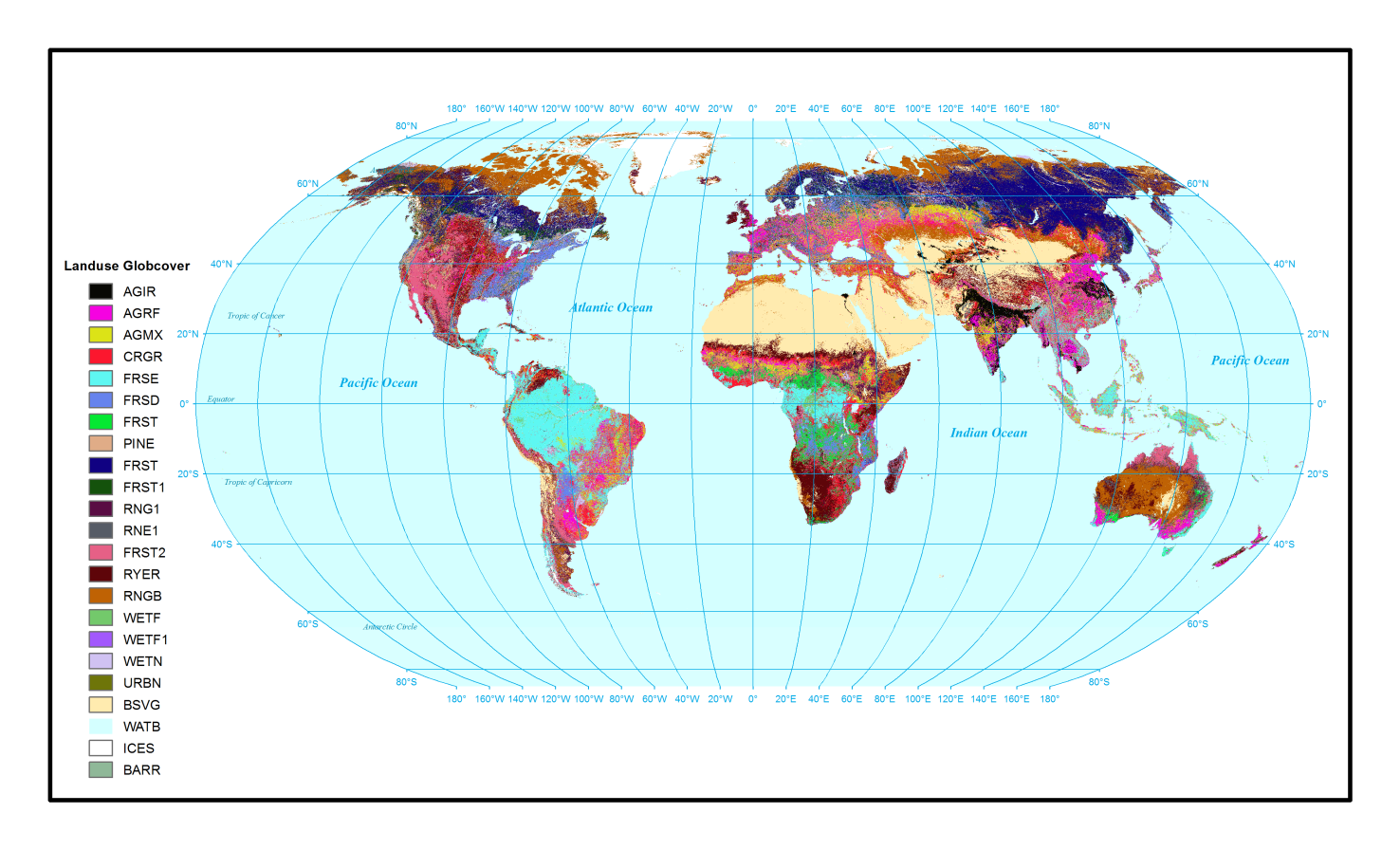


**Figure S4.** Unique landcover units in the Global_Landuse_Globcover.


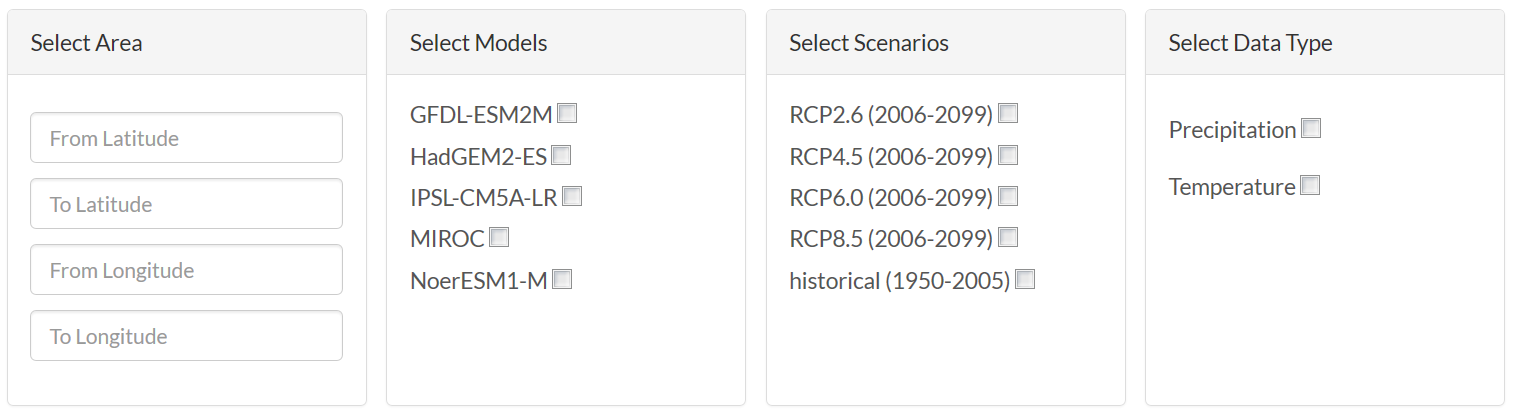


**Figure S5.** Template for downloading GCM data.


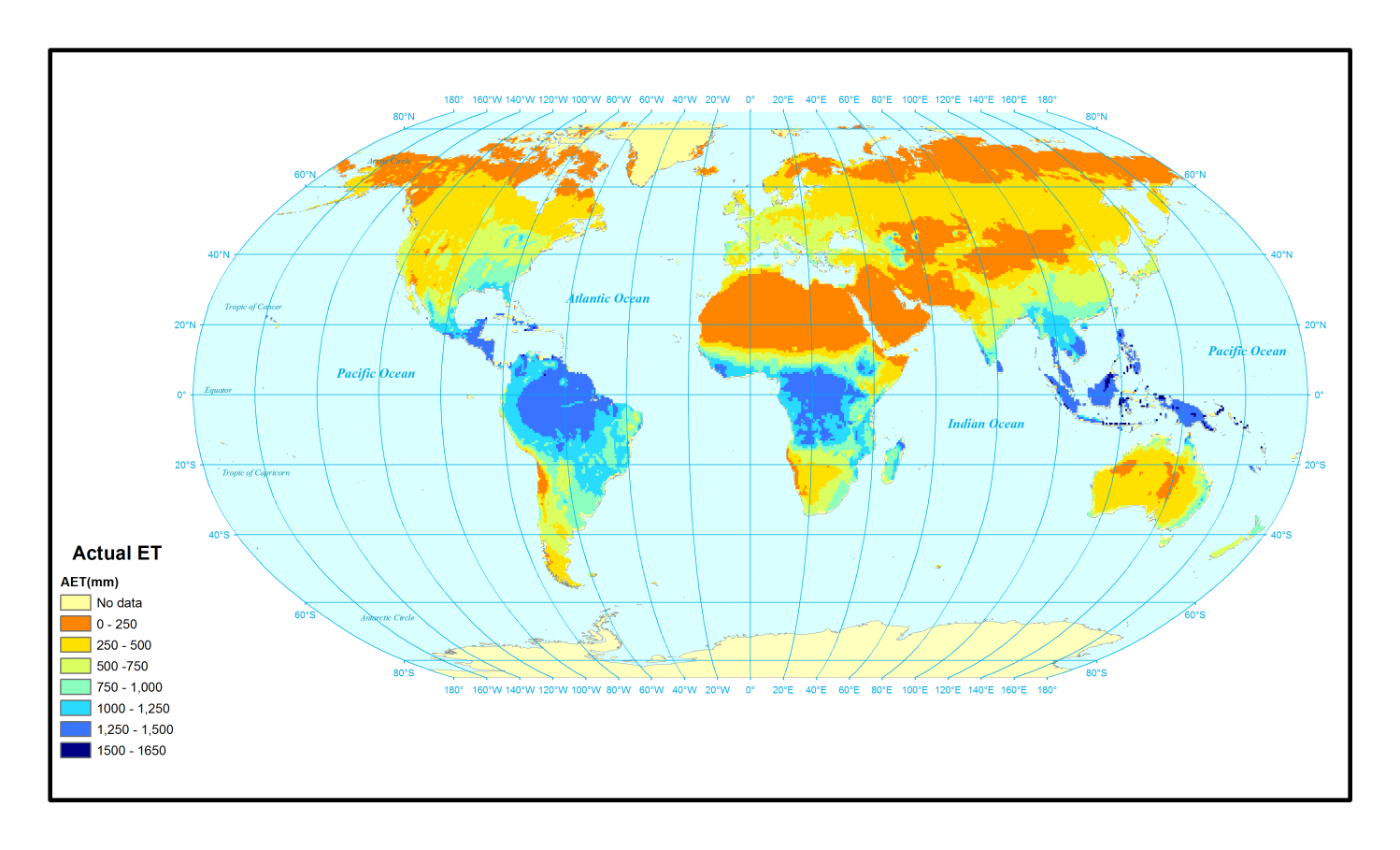


**Figure S6.** Spatial map of average annual (1983-2006) land surface actual

evapotranspiration (AET) (mm y^-1^).
